# The early discontinuation of adjuvant hormone therapy is associated with a poor prognosis in Japanese breast cancer patients

**DOI:** 10.1007/s00595-013-0762-7

**Published:** 2013-10-19

**Authors:** Kenji Taketani, Eriko Tokunaga, Nami Yamashita, Kimihiro Tanaka, Sayuri Akiyoshi, Satoko Okada, Koji Ando, Yasue Kimura, Hiroshi Saeki, Eiji Oki, Masaru Morita, Tetsuya Kusumoto, Yoshihiko Maehara

**Affiliations:** 1 Department of Surgery and Science, Graduate School of Medical Sciences, Kyushu University, 3-1-1 Maidashi, Higashiku, Fukuoka 812-8582 Japan; 2 Department of Comprehensive Clinical Oncology, Graduate School of Medical Sciences, Kyushu University, 3-1-1 Maidashi, Higashiku, Fukuoka 812-8582 Japan

**Keywords:** Adherence, Early discontinuation, Adjuvant hormone therapy, Side effect

## Abstract

**Purpose:**

It is important for patients to complete the planned hormone therapy to reduce both the recurrence and mortality rates of hormone receptor-positive breast cancer. We investigated the rates and factors related to the early discontinuation of adjuvant hormone therapy at our institution.

**Methods:**

We identified 145 females prescribed adjuvant hormone therapy who were followed up for longer than 5 years. The rate of completing the planned hormone therapy and factors related to early discontinuation were examined. The relapse-free survival rate was examined between the completion group and the discontinuation group.

**Results:**

The completion rate was 90.6 %. The primary reason for discontinuing hormone therapy within 5 years was side effects, such as arthritic pain. The primary factor related to early discontinuation was a significantly younger age. The relapse-free survival rate was significantly lower in the discontinuation group (*p* = 0.025).

**Conclusions:**

More than 90 % of the patients completed the planned adjuvant hormone therapy, and early discontinuation was related to a shorter RFS. To improve the rate of the successful completion of adjuvant hormone therapy, it is important to provide supportive care to reduce the occurrence of side effects and to care for young females with a desire to become pregnant.

## Introduction

Treatment with postoperative hormone therapy reduces the recurrence and mortality of early hormone receptor (HR)-positive breast cancer in both pre- and postmenopausal females [1]. Oral hormone therapies include selective estrogen receptor modulators (SERMs) and aromatase inhibitors (AIs), which are typically prescribed for 5 years or longer. Among premenopausal females with postoperative HR-positive early breast cancer, treatment with tamoxifen (TAM) improves the disease-free survival compared with that observed in patients receiving no adjuvant treatment [2]. In addition, inhibiting the ovarian function with LHRH agonists has been proven to reduce the recurrence rate of breast cancer [3]. A meta-analysis of randomized trials of TAM in patients with early breast cancer demonstrated significant 15-year risk reductions in cancer recurrence and mortality [4]. In the ATLAS (Adjuvant Tamoxifen: Longer Against Shorter trial), 10 years of TAM treatment was found to be related to a superior prognosis compared to that observed following 5 years of treatment [5]. Among postmenopausal females, treatment with AIs improved the disease-free survival compared with that achieved with TAM [6, 7]. For perimenopausal females, switching adjuvant hormone therapy from TAM to AIs is thus considered to be an effective treatment strategy [8–10].

The lack of adherence to prescribed medications is a well-known problem in the medical literature [11]. Many patients fail to fill the initial prescription (noninitiation), take the drug on a daily basis as prescribed (nonadherence) or continue long-term treatment with the drug (early discontinuation) [11]. Among patients receiving adjuvant hormone therapy, 32 % discontinue therapy by 4.5 years, and of those who continue, only 72 % are fully adherent [12]. The adherence and discontinuation of SERMs and AIs are related to higher recurrence rates and worse survival in many studies [13, 14]. The discontinuation rate for TAM and AIs is approximately 7–10 % per year [13, 15–21]. In clinical trials, 8–28 % of patients do not complete the treatment as recommended [6, 22, 23]. Moreover, reports have indicated that the rate of completing the recommended hormone therapy ranges from only 10–50 % due to failure to take the correct dose at the prescribed frequency or due to the discontinuation of therapy in both clinical trials and the clinical practice setting [18, 24–28]. A consistent finding in the literature is that treatment side effects are strongly associated with adherence to or continuation with adjuvant therapy [29]. With regard to the factors predicting the adherence to hormone therapy regimens, Hershman et al. [14] reported that Asian race, being married and a longer prescription refill interval are associated with higher rates of completion of therapy.

Although there have been many reports regarding adherence to and/or the completion of hormone therapy in European countries and the United States, no studies of Japanese breast cancer patients have so far been reported. It is important to verify whether the same factors identified in these studies apply to the Japanese breast cancer patients [30].

In the current study, we investigated the rates of completion and factors related to the early discontinuation of adjuvant hormone therapy and compared the prognoses between the completion group and the discontinuation group among Japanese breast cancer patients.

## Patients and methods

### Patient selection and study design

This study was a retrospective observational study. Among the patients who underwent curative surgery at the Department of Surgery and Science, Kyushu University Hospital between 2002 and 2006, we selected 263 patients with breast cancer of stage I–III according to the UICC TNM classification. Written informed consent regarding data acquisition was obtained from all patients before surgery. The rate of and reason for discontinuing adjuvant hormone therapy and the relationships between these factors and the prognosis were investigated by reviewing the patients’ medical records. The medical records contained information regarding the status of recurrence, date of last follow-up, time after surgery and clinical characteristics, such as the stage, type of operation, history of cancer therapy, nuclear grade and the expression of hormone receptors (HRs) [the estrogen receptor (ER) and progesterone receptor (PR)] and the HER2 status.

### Evaluation of the ER, PR and HER2 status

The ER, PR and HER2 status was evaluated as described previously [31]. The ER and PR were considered to be positive if ≥1 % of the nuclei of the tumors were stained during IHC. The tumors were considered to be HER2-positive if they were scored as either 3+ on IHC or 2+ on IHC with HER2 amplification (ratio > 2.0) based on fluorescence in situ hybridization.

### Statistical analysis

The survival analyses were performed using the Kaplan–Meier method, and differences between the groups with regard to the survival and relapse-free survival times were evaluated using the log-rank test. All of the analyses were conducted using the JMP version 9 software program (SAS Institute Inc., Cary, NC). Differences were considered to be significant at values of *p* < 0.05.

## Results

### Rate of and reason(s) for discontinuing adjuvant hormone therapy

We identified 263 females diagnosed with stage I–III breast cancer who had undergone surgery between January 1, 2002 and December 31, 2006 in our department. One hundred and seventy-seven patients were diagnosed with HR-positive breast cancer, while the remaining 86 patients were diagnosed with HR-negative breast cancer. Among the HR-positive patients, 171 were treated with adjuvant hormone therapy. SERMs and AIs were prescribed to 103 (71 %) and 42 (29 %) patients at the start of adjuvant therapy, respectively.

**Fig. 1:**
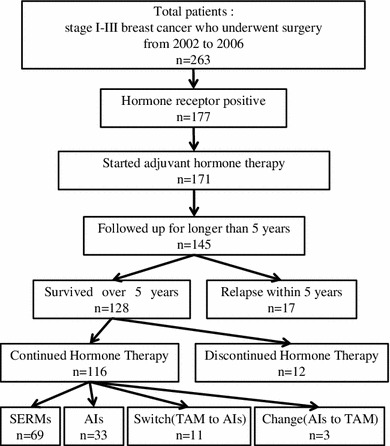
A schematic diagram of the patients evaluated in this study. Among the 145 patients followed up for longer than 5 years, 128 survived longer than 5 years without relapse. A total of 116 patients continued hormone therapy for 5 years, while 12 patients discontinued therapy. In the continuation group, 69 patients were treated with SERMs, 22 patients were treated with AIs, 11 patients were switched from TAM to an AI and three patients were switched from an AI to TAM

Among these patients, 26 were excluded from the study due to failure to participate in follow-up as a result of transferring to another hospital or ending hospital visits with no contact (Fig. 1). One hundred and forty-five patients who were followed up for longer than 5 years were included in the analysis. Seventeen patients who experienced relapse within 5 years changed therapies.

The rate of completing the planned hormone therapy and the factors related to the early discontinuation of hormone therapy were examined among the 128 patients who survived longer than 5 years without relapse. Twelve (9.4 %) patients discontinued and 116 (90.6 %) patients completed the planned hormone therapy. In the completion group, SERMs and AIs were administered to 69 (59.4 %) and 33 (28.4 %) of the patients for 5 years, respectively. In addition, 11 (9.4 %) perimenopausal patients switched from TAM to AI after menopause. There was a change from an AI to TAM in three (2.6 %) cases due to the development of arthritic pain (Fig. 1).

In the discontinuation group, the reasons for discontinuing hormone therapy within 5 years included side effects, such as arthritic pain, in five (41.7 %) cases, a desire to become pregnant in two (16.7 %) cases, dependence on Qigong in one (8.3 %) case and no specific reason in four (33.3 %) cases (Table 1). We found that females younger than 40 years of age discontinued adjuvant hormone therapy significantly more frequently than did older patients (Table 2). Two of the four patients under age 40 (50 %) wished to become pregnant.

**Table 1:** The reasons for the discontinuation of hormone therapy

Reason	Number (%)
Side effect	5 (41.7)
Arthritic pain	2 (16.7)
Headache	1 (8.3)
Endometrial hypertrophy	1 (8.3)
Atypical genital bleeding	1 (8.3)
Desire to be pregnant	2 (16.7)
Taking Qigong	1 (8.3)
No specific reason	4 (33.3)

**Table 2:** The clinicopathological features of the completion and the discontinuation groups

Factors	Completion group	Discontinuation group	*p* value
	(*n* = 116)	(*n* = 12)	
Age (years)			
<40	7	4	0.001
>40	109	8	
T			
1.2	96	10	0.959
3.4	20	2	
N			
Negative	83	10	0.383
Positive	33	2	
NG			
1	73	8	0.833
2	26	3	
3	17	1	
Ly			
Negative	88	9	0.947
Positive	28	3	
Unknown	1	0	
v			
Negative	113	12	0.645
Positive	2	0	
Unknown	1	0	
Surgery			
Mastectomy	57	8	0.247
Lumpectomy	59	4	
ER			
Negative	2	0	0.646
Positive	114	12	
PR			
Negative	23	2	0.792
Positive	93	10	
HER2			
Negative	49	7	0.321
Positive	64	5	
Unknown	3	0	
Chemotherapy			
+	28	2	0.56
−	88	10	
Radiation			
+	54	5	0.746
−	62	7	

### Relationship between the discontinuation of adjuvant hormone therapy and the prognosis

**Fig. 2:**
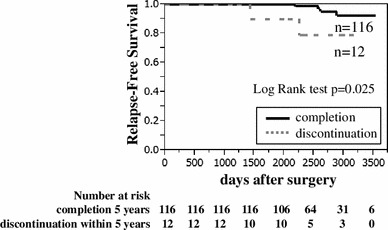
Kaplan–Meier curves comparing the RFS rates in the continuation and discontinuation groups. The continuation group exhibited a significantly better prognosis than the discontinuation group

We classified the patients into two groups: the continuation group and the discontinuation group. We analyzed the overall survival (OS) and relapse-free survival (RFS) rates between the two groups. There were no significant differences in the OS between the two groups. However, the RFS rate was significantly lower in the discontinuation group (*p* = 0.025) (Fig. 2).

## Discussion

To the best of our knowledge, this is the first report regarding the completion and discontinuation of adjuvant hormone therapy in Japanese breast cancer patients. With regard to breast cancer therapy, both surgery and adjuvant therapy are important factors associated with improvements in the survival rate. The mortality rate has been improved with the administration of hormone therapy to patients with HR-positive breast cancer [1]. Currently, cancer research is focused on discovering and proving the efficacy of new interventions to reduce cancer mortality. However, these treatments are meaningless if the patient does not take the medication as planned.

In previous clinical trials, the rate of completing hormone therapy among ER-positive breast cancer patients has been reported to be 8–28 % [6, 22, 23, 32]. On the other hand, in clinical practice, the discontinuation rate has been reported to be 31–73 % [33]. The low completion rate is considered to be largely due to differences in provider support, that is, who is responsible for providing cancer follow-up care [33]. The Behavioral Risk Factor Surveillance System demonstrated that only 20 % of all cancer survivors continue to see an oncologist or cancer specialist as their primary provider for cancer follow-up care. Compared with primary care physicians (PCPs), oncologists are less likely to believe that PCPs have the skills to conduct appropriate testing to detect breast cancer recurrence (59 vs. 23 %, *p* < 0.001) or provide care for the late effects of breast cancer (75 vs. 38 %, *p* < 0.001)[34].

In this study, it was difficult to evaluate the precise level of adherence to the hormone therapy regimen in each patient based on a review of the medical records. Therefore, only the rate of completion or early discontinuation was evaluated in this study. There is little knowledge regarding the factors related to adherence. Smoking, the breast cancer risk, extremes of age, a non-white ethnicity, the socioeconomic status and the level of education are all associated with the adherence to TAM treatment as adjuvant therapy [35]. Another report showed that a younger or older age, the use of lumpectomy or unknown surgery and the presence of additional comorbid conditions are associated with the discontinuation of hormone therapy. On the other hand, an Asian/Pacific Islander ethnicity, being married, an earlier age at diagnosis, prior receipt of adjuvant chemotherapy, receipt of adjuvant radiation therapy and a longer prescription refill interval have been found to be associated with the completion of 4.5 years of hormonal therapy [12]. Similar phenomena have been observed for other oral medications, such as Gleevec, used to treat gastrointestinal stromal tumors or chronic myelogenous leukemia [36, 37].

The side effects are strongly associated with the adherence to adjuvant therapy, and reducing and controlling side effects are one way to increase the adherence rate. In a survey of 622 postmenopausal females, 30 % of the patients discontinued hormone therapy, and the rate of discontinuation related to side effects was reported to be very high (84 %) [38]. In the present study, side effects, such as arthritic pain, were also a major factor associated with the discontinuation of hormone therapy (41.7 %). In addition, the younger females exhibited early discontinuation of adjuvant hormone therapy significantly more often in our study. Half of these patients discontinued treatment because they wished to become pregnant. Identifying and reducing the reasons for nonadherence and discontinuation of oral hormone medications can increase adherence and the completion rate, and ultimately improve the outcomes. Therefore, clinicians must address side effects.

Figure 2 shows the importance of the effects of hormone therapy on late relapse. The EBCTCG (Early Breast Cancer Trialists’ Collaborative Group) showed that the risk reduction effect of endocrine therapy was seen even 15 years after the initiation of TAM in estrogen receptor-positive breast cancer [4]. This is considered to be a carryover effect. It is thought that this carryover effect was reduced in the discontinuation group compared to the completion group in this study.

In conclusion, we demonstrated that, in Japan, the rate of completing adjuvant hormone therapy in HR-positive breast cancer patients is higher than that reported in other countries. Side effects and a younger age were the major factors associated with early discontinuation, while early discontinuation was significantly associated with a shorter RFS. Therefore, we emphasize that controlling side effects and caring for young females are important for helping patients to continue and complete their planned adjuvant hormone therapy.
